# Heterogeneous Surfaces as Structure and Particle Size Libraries of Model Catalysts

**DOI:** 10.1007/s10562-018-2506-1

**Published:** 2018-07-31

**Authors:** Yuri Suchorski, Günther Rupprechter

**Affiliations:** Institute of Materials Chemistry, Technische Universität Wien, Getreidemarkt 9, 1060 Vienna, Austria

**Keywords:** Heterogeneous catalysis, Surface reaction kinetics, Photoemission electron microscopy, Processes and reactions, Hydrogen–oxygen reaction

## Abstract

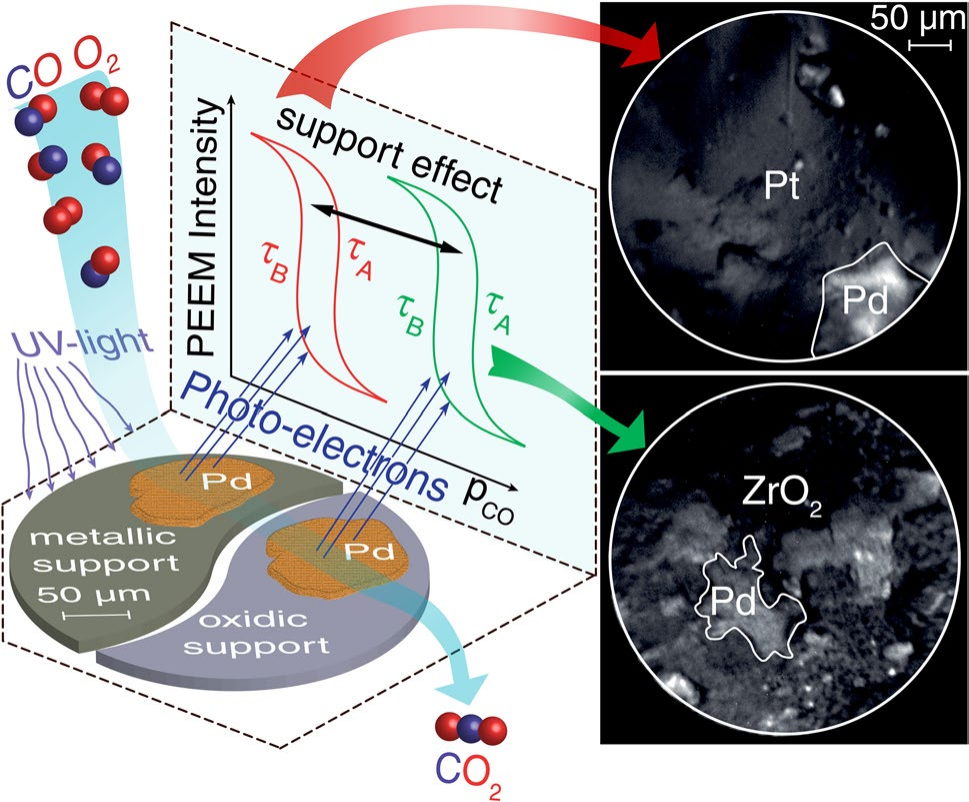

## Heterogeneous Catalysis and Structure Libraries

**Fig. 1:**
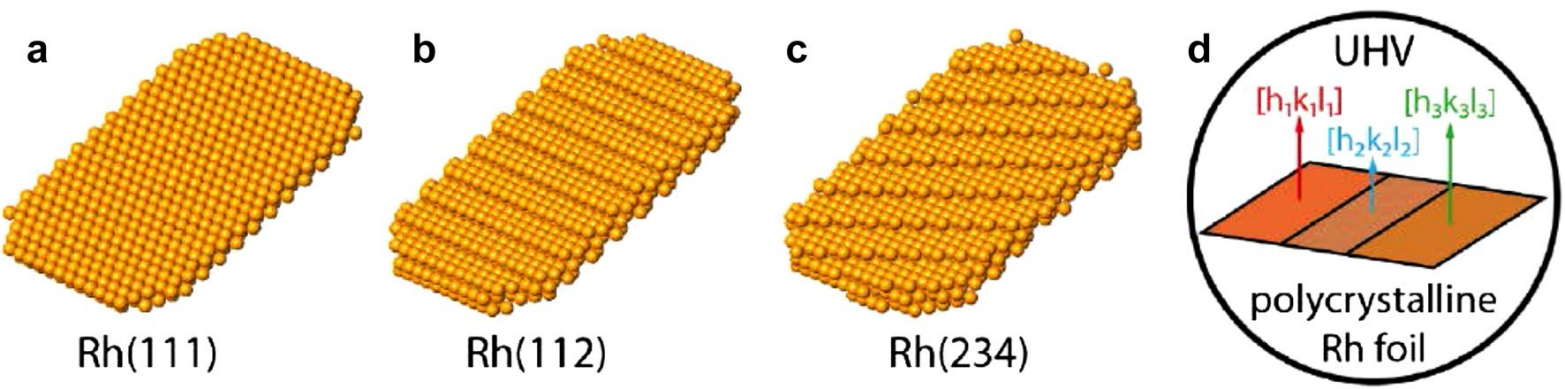
Physical, chemical and catalytic properties depend on the particular surface structure: **a** crystallographic structure of an fcc Rh(111) surface; **b** the same for a stepped Rh(112) surface; **c** the same for a “kinked” (234) surface; **d** the idea of a surface structure library

Heterogeneous catalysis plays a decisive role in the current and, even more, in the future production and development of sustainable and environmentally neutral energy sources, fine chemicals and pharmaceuticals [1]. The catalytic properties of heterogeneous catalysts, frequently based on precious metals such as Pt, Pd, Rh or Ir, are strongly dependent on their surface structure: atomically smooth surfaces, such as a (111)-oriented Pt surface (Fig. 1a), are often less reactive than “rough”, especially stepped (Fig. 1b) or “kinked” (Fig. 1c) surfaces of the same metal. This finding originates mainly from “surface science” (surface analytical) investigations of crystallographically differently oriented surfaces of cm-sized single crystals (Fig. 1a–c) in ultra-high vacuum (UHV; p < 10^−8^ mbar) or high vacuum (HV; p < 10^−3^ mbar) (Refs [2–5] and references therein).

However, comparing the catalytic activity of various single crystal surfaces (e.g. (111), (100), (110), (112), (234), etc) of a specific (precious) metal for a certain reaction can be very time-consuming. Furthermore, a comparison that works well under UHV may become more difficult under HV reaction conditions: for a true quantitative comparison, identical reactor pressures, identical temperatures and identical temperature ramps must be guaranteed, placing high demands on experiments.

A solution of this problem is to generate a kind of “surface structure library” (Fig. 1d), i.e. to construct a sample containing adjacent regions with different crystallographic orientation. However, these “adjoining single crystals” must also be sufficiently decoupled from each other, so that their inherent catalytic behaviour can still be determined.

**Fig. 2:**
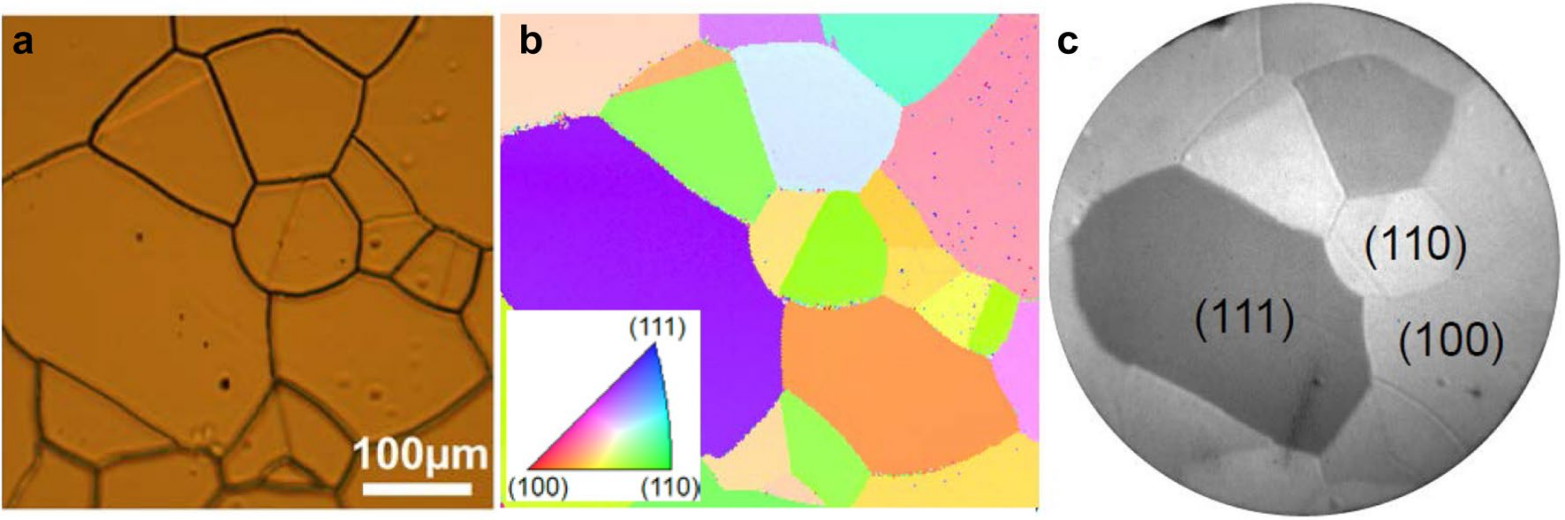
Polycrystalline Pd foil as surface structure library in catalysis: **a** optical microphotography of a 500 × 500 µm^2^ region; **b** EBSD image of the same region; the EBSD color code is shown in the inset; **c** the same region, imaged with PEEM, individual crystallographic orientations are marked. **b** and **c** Adapted from Ref. [10] https://pubs.acs.org/doi/abs/10.1021/jp312510d with permission from the American Chemical Society

For realization, single crystals can be cut and polished such that two or three vicinals are exposed simultaneously, but the fabrication is expensive and the samples are difficult to use. In contrast, polycrystalline metal foils, e.g. of rhodium (Fig. 1d) or palladium (Fig. 2a), are significantly cheaper and consist of many more µm-sized, crystallographically differently oriented Rh(hkl) or Pd(hkl) domains separated by grain boundaries, which makes them real surface structure libraries [6–10]. The crystallographic orientation of the individual domains can be determined by electron backscatter diffraction (EBSD) (Fig. 2b). Since the crystalline grains are in the 10–300 µm range, hundreds of individual domains with dozens of different orientations can be found on a cm-sized sample.

During examination of a surface reaction, all the different domains are then “automatically” exposed to the very same pressure and temperature conditions, but the determination of specific catalytic properties requires spatially-resolved surface analysis methods. Photoemission electron microscopy (PEEM), first used by Nobel Laureate Ertl to visualize catalytic reactions in situ, is well suited for the spatially-resolved monitoring of surface reactions [11].

For such monitoring, the catalyst sample is irradiated with ultraviolet light (UV) during the gas/surface interaction and the released photoelectrons form a real time image of the sample surface, with the image contrast being determined by the local work function (Fig. 2c) [6, 7, 10]. However, to apply microscopy to study orientation-specific local catalytic properties, a relationship between the surface image and the reaction kinetics must be established.

## Reaction Kinetics by Imaging

The use of polycrystalline structure libraries raises the question of how local (spatially-resolved) kinetic measurements can be carried out on the µm scale. For investigations on single crystals (homogeneous surfaces), mass spectrometry (MS) is mainly used to detect reaction products; no spatial resolution is required since the local activity is equal to the activity averaged over the entire uniform single crystal. In case of spatially heterogeneous model systems, such as polycrystalline foils, MS is limited by its averaging principle: MS can not distinguish between the reaction products originating from different regions of the sample. Although a thin capillary for local gas sampling could be used, such capillary has to scan over the sample (“scanning-MS”) to examine different regions [12]; this prevents, however, simultaneous (parallel) measurements of different regions, a drawback of every scanning process.

**Fig. 3:**
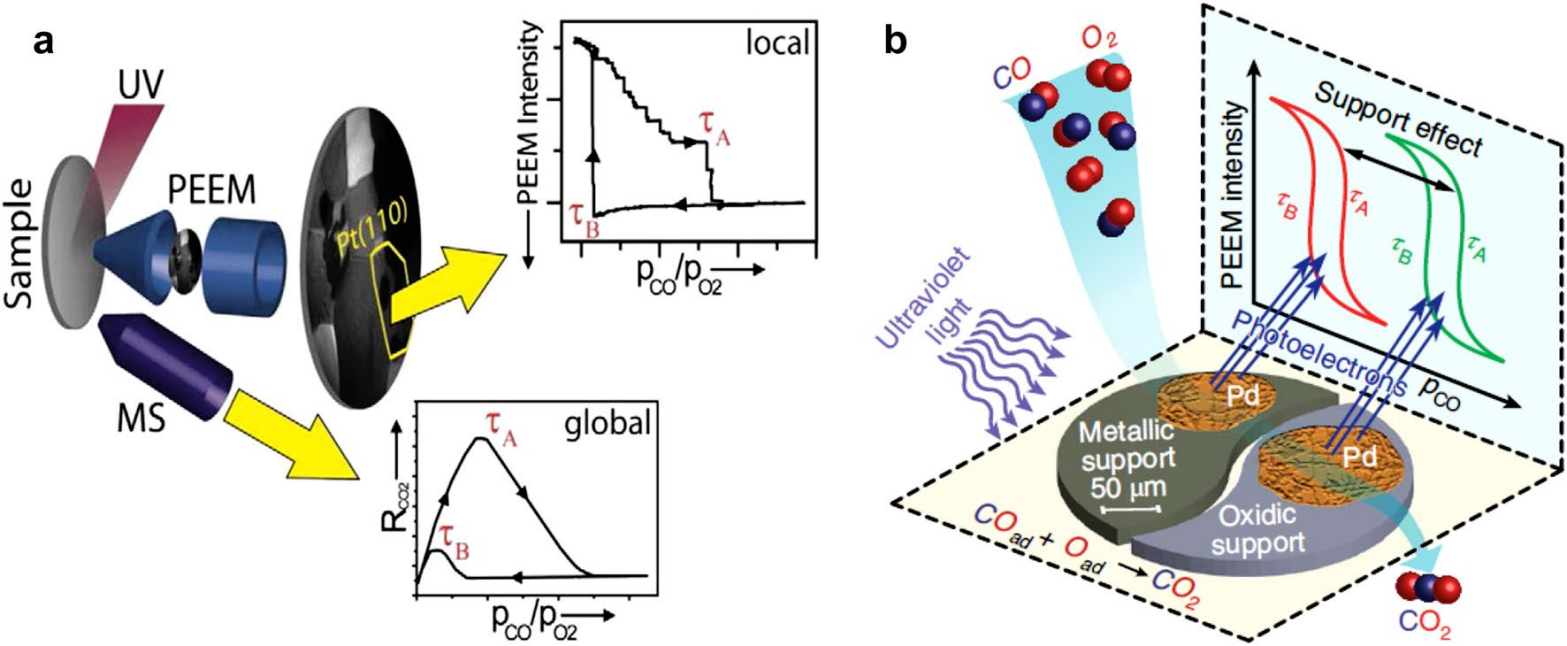
Concept of local reaction kinetics by imaging. **a** Catalytic reaction on a polycrystalline foil is monitored in situ, simultaneously by PEEM and MS. Local data obtained from the intensity analysis of PEEM video-sequences for each individual (hkl)-domain are compared with global (averaged) MS data; **b** comparison of kinetic behaviour of individual “quasi-unsupported” and oxide-supported Pd particles. **a** Reproduced from Ref. [7] with permission from Springer, **b** reproduced from [38] with permission from Springer Nature

In turn, microscopes, which are based on a parallel imaging principle, collect information simultaneously from few extended regions and, in addition, techniques such as PEEM are able to distinguish between a clean (adsorbate-free) and reactant-covered surface: e.g. between the adsorbate-free, CO-covered and O-covered regions in catalytic CO oxidation [11, 13]. This specific ability was the basis of numerous qualitative PEEM investigations of CO oxidation on platinum single crystal surfaces [11, 13, 14]. In addition, a direct correlation between the work function of a CO- or O-covered Pt single crystal surface and its spatially-averaged catalytic activity in CO oxidation was established [15]. This correlation can be extended to the (work function determined) PEEM image intensity. The same work function/activity correlation must be also valid “locally”, i.e. down to the nm range, thus we have employed it for our “local kinetics by imaging” approach, in which kinetic information is derived exclusively from the local PEEM image intensity (depending on the local work function [6, 16]). The basic concept is illustrated in Fig. 3a for the exemplary catalytic CO oxidation on Pt: during the ongoing reaction, the global rate of CO_2_ production (global reaction kinetics; averaged over the entire sample) is (conventionally) recorded by a mass spectrometer. At the same time, PEEM visualizes the reaction in situ with spatial resolution. The digital evaluation of the recorded video sequences then reveals the kinetics of the reaction on individual, different domains of the polycrystalline sample. Figure 3b illustrates applying this approach to the comparison of the kinetic behaviour of individual “quasi-unsupported” and oxide-supported Pd particles in CO oxidation. These and other recent applications of “kinetics by imaging” are discussed below.

## Catalytic Ignition and Kinetic Transitions

Emissions of internal combustion engines are still the main obstacle of urban mobility and, independently of the current “hot” NO_x_ discussion, the concentration of harmful CO must be strictly limited. The three-way automotive catalytic converter has a great success story, but the progress in stop-start systems and hybrid technology has created new issues. Stopping the engine at traffic lights or in traffic jams leads to rapid cooling of the catalytic converter, and at every new “cold start” the produced CO can not be efficiently oxidized. The same holds for intermissions in the operation of the combustion engine of a hybrid drive. It is therefore essential to minimize emissions especially during the starting period. Sophisticated processes have been developed, such as electrically preheating the catalyst to reach the critical “ignition temperature” earlier, lean mixture operation, exhaust afterburners, secondary air systems, etc. [17].

Alternatively, lowering the critical “ignition temperature” provides a cost-effective and energy-neutral solution. Very often, catalytic ignition is treated as a pure thermal balance problem, namely, as the point when the heat generated by the exothermic reaction exceeds the dissipated heat [18]. However, catalytic ignition is a complex convolution of reaction kinetics and heat generation, since the generated heat is determined by the reaction rate, which in turn is governed by the reaction kinetics. For CO oxidation, the temperature-controlled kinetic transition from the stationary state of low activity to that of a high catalytic activity determines the ignition temperature [19]. Since such a transition depends on the nature and surface structure of the catalyst, it is interesting to compare different metals and different crystallographic orientations, in order to develop strategies for reaching the lowest possible ignition temperatures.

**Fig. 4:**
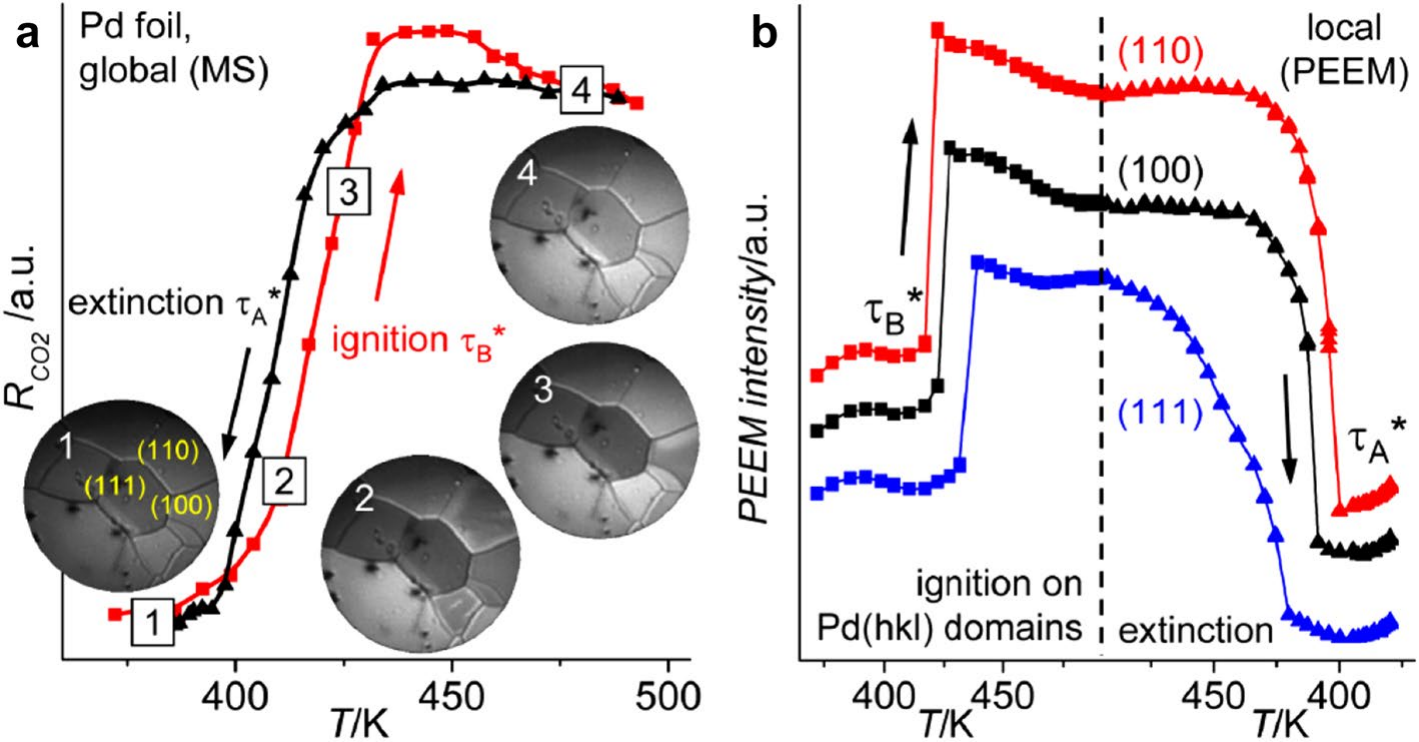
Catalytic ignition of CO oxidation on crystallographically differently oriented domains of a polycrystalline Pd foil: a) Ignition (red squares) and extinction curves (black triangles), as CO_2_ production rate, measured globally by MS, at cyclic variation of sample temperature (0.5 K s^−1^) at constant p_CO_ = 5.8 × 10^−6^ mbar and p_O2_ = 1.3 × 10^−5^ mbar. The simultaneously recorded PEEM video-sequences illustrate the ignition process: (1) inactive, CO-covered surface; (2) ignition starts on the (110) domains; (3) ignition continues on the (100) domains; (4) oxygen-covered, active surface. **b** Spatially-resolved ignition/extinction measurements: local PEEM intensity of the individual (110), (100) and (111) domains during the same cyclic temperature variations as in (**a**). The vertical dashed line marks the reversal from heating to cooling. Reproduced from Ref. [9] with permission from John Wiley and Sons

By applying the *reaction kinetics by imaging* approach to individual (hkl) domains of polycrystalline Pd and Pt foils, we were able to compare different surface orientations of the two metals under identical reaction conditions, providing access to their inherent reaction behavior [6, 9, 10]. Figure 4 shows the global (Fig. 4a) and local (Fig. 4b) ignition curves of CO oxidation on Pd for a temperature range of 370–500 K, at constant CO and O_2_ pressure. The global CO_2_ rate rises steeply between 380 and 440 K, marking the transition τ_B_ from the inactive steady state (CO poisoned surface, video-frame 1 in Fig. 4a, dark contrast) to the active steady state when the surface is oxygen covered (frame 4, bright contrast). Similar to the global MS signal of the total CO_2_ rate, the locally-resolved PEEM intensities of the various domains show “jumps”, characterizing the local kinetic transitions (i.e. ignitions) on the individual grains of the Pd foil (Fig. 4b). These transitions do not occur simultaneously for different orientations, but show a distinct structure dependence: critical temperatures of 417 K for Pd(110), 423 K for Pd(100) and 432 K for Pd(111) can be clearly identified. The same holds true for the “extinction” of the reaction, i.e. the transition τ_A_ from the active steady state to the inactive state, when the sample is cooled down.

The curve of the MS-measured global CO_2_ production rate appears “less sharp” (black curve in Fig. 4a) than the local (independent) extinction curves of the individual grains (Fig. 4b). This demonstrates the limits of spatially-averaged methods, such as MS, which can not reveal the peculiarities of local kinetics. In contrast, the spatially-resolved PEEM can use the full bandwidth of a surface structure library, in this case a polycrystalline Pd foil, for decoding the structure dependence of catalytic ignition. In addition, the method allows a direct comparison of different metals: in a “line-up” with Pt, Pd is the catalyst with significantly higher CO tolerance (cf. Ref. [9]).

## Singing in a Choir, But Each Domain with Its Own Voice

Self-sustaining oscillations are a fascinating phenomenon associated with self-organization, observed in biology, chemistry, physics, sociology, and even economics. In simple terms, a certain property/parameter varies periodically despite constant external conditions. Examples of such oscillations range from fluid mechanics [20] to ecosystems (e.g., described by classic Lotka–Volterra predator–prey models [21]) to the real estate markets [22]. In chemistry, self-sustaining oscillations occur as periodically oscillating reaction rates, despite constant external conditions (gas pressure, temperature). More than one hundred reactions with oscillating reaction kinetics under stationary conditions are established, in particular the Belousov–Zhabotinski [23] and Bray–Liebhafski (chemical clock, [24]) reactions.

In heterogeneous catalysis, self-sustaining oscillations were first observed in the 1970s upon CO oxidation on Pt surfaces [25] and in the 1980s for catalytic NO reduction [26]. Since then, oscillating surface reactions have evolved into a fast developing topic, even with several technological applications [27]. Apart from oscillations in reaction rate, reactants can also redistribute on the catalytic surface, forming repeating concentration patterns, which propagate as chemical waves. This behaviour can be described by nonlinear reaction dynamics, a contemporary research field, highlighted in 2007 by the Nobel Prize for Ertl [11].

To date, such concentration patterns have been mainly observed on single crystal surfaces, with the oscillation frequency being the same over the entire uniform sample surface [11, 14, 28, 29]. Using a polycrystalline (Rh) metal foil as surface structure library, a new phenomenon was recently discovered, namely multifrequential oscillations during catalytic hydrogen oxidation [30]. Every specific crystallographic surface structure, i.e. every particular Rh domain, formed an individual spiral pattern with a specific oscillation frequency, despite the global diffusion coupling.

**Fig. 5:**
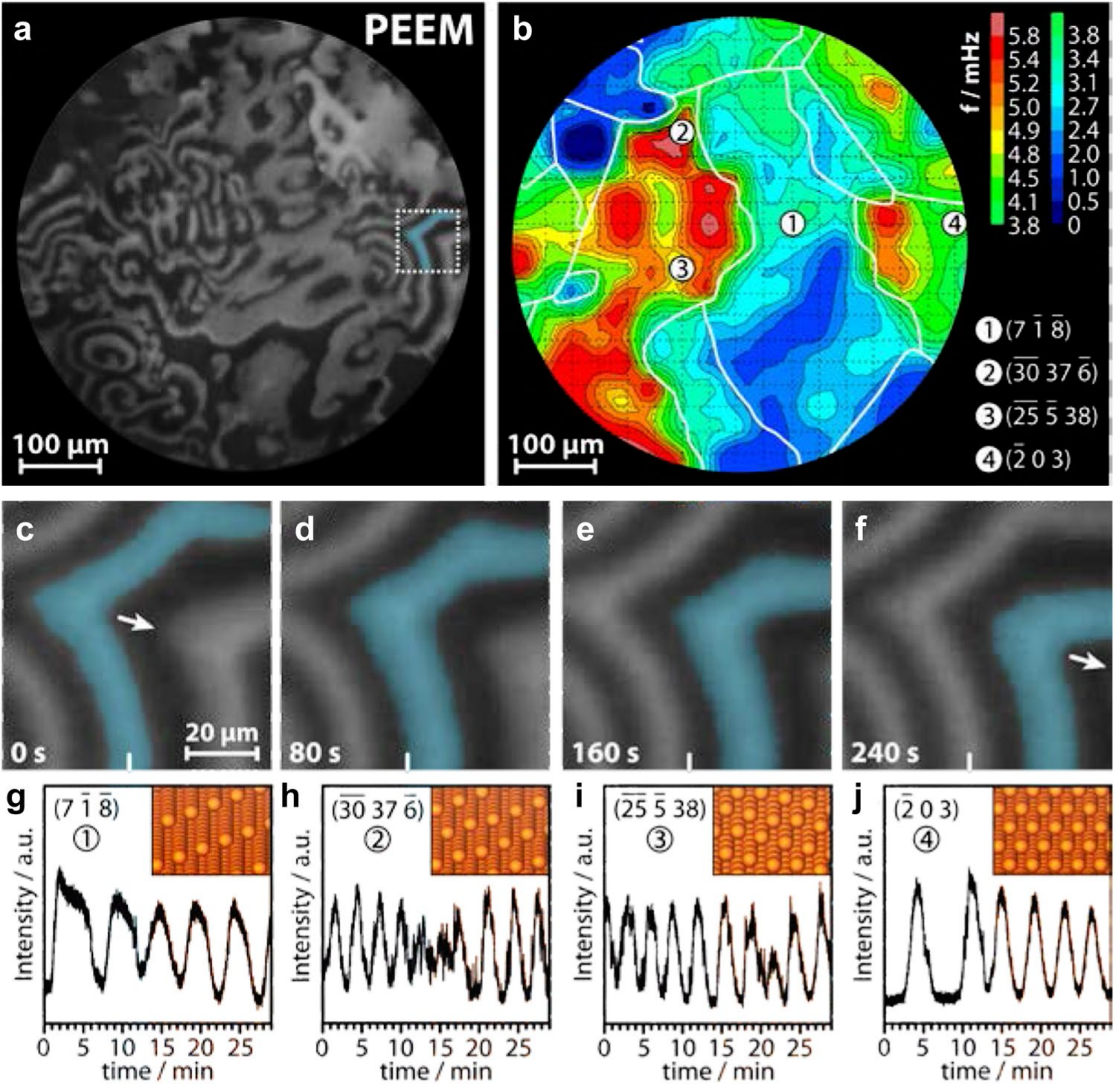
Isothermal kinetic oscillations of H_2_ oxidation on a polycrystalline Rh foil: **a** PEEM snapshot taken during H_2_ oxidation at constant p_O2_ = 1.1 × 10^−6^ mbar, p_H2_ = 8.4 × 10^−7^ mbar and T = 433 K; **b** “frequency landscape image” of the observed oscillations. Crystallographically different domains are marked by white lines. The round number symbols indicate selected crystallographic orientations; **c**–**f** propagation of a chemical wave in the section marked in (**a**); **g**–**j** time-dependent (oscillating) local PEEM intensities of selected regions. The positions of the corresponding regions of interests (diameter 1 µm) are marked by the number symbols in (**b**). The inserts in **g**–**j** show the ball models of the corresponding surface structures. The particular orientation of the individual domains (Miller indices) was determined by EBSD. Reproduced from Ref. [30] with permission from Springer Nature

Figure 5a shows a PEEM video-frame recorded in situ during H_2_ oxidation on polycrystalline Rh at constant H_2_ and O_2_ pressures and temperature.

The dark areas correspond to the oxygen-covered inactive Rh surface and the bright areas to the catalytically active surface with low hydrogen coverage. Such surface pattern, visualized by PEEM, does not remain static but exhibits a complex turbulence-like “living” surface, consisting of repeating “spirals” that propagate as chemical waves (enlarged view in Fig. 1c–f; one wave was coloured). It is interesting that the “rotation speed” of the spirals varies significantly for different surface orientations.

In the PEEM image, the brightness of different crystallographic domains (Fig. 5a, b) reflects the local reaction rate (*kinetics by imaging*, [16]) meaning in the present case that high image brightness corresponds to the active state (high reaction rate) and the low image brightness to the inactive state (low reaction rate). In this way, the local oscillation frequencies in the reaction rate can be determined from PEEM image-brightness analysis at selected positions (Fig. 5g–j; for larger ball-models refer to Supplementary Fig. 1 of Ref. [36]). Since the crystallographic orientation of the individual domains is known from EBSD (see labels in Fig. 5b), the local oscillation frequencies can be correlated with the local surface structure of the corresponding domains. In this way, the measured local oscillation frequencies can be displayed as a “frequency map” in Fig. 5b.

Generally, a surface reaction can only show oscillating behavior when a feedback mechanism is present, in addition to the bistability of the reaction [8, 27]. Bistability describes the existence of two alternative stationary states (active or inactive in this case) under identical external conditions, so that the state of the system is determined by its previous history. The feedback mechanism periodically switches between these two stationary states, e.g. by varying the adhesion coefficient of the reactants by changing the surface structure [11, 14, 29], by forming and depleting “subsurface” oxygen [14, 29] or by oxide formation [31].

The decisive role of stepped Rh surfaces in the described investigation, together with the fact that oscillating H_2_ oxidation had never been observed on smooth pure Rh surfaces, suggests that the periodic formation of subsurface oxygen acts as the feedback mechanism. The PEEM observations did not provide direct evidence for the formation of subsurface oxygen, but it has been reported that on the smooth Rh(111) surface subsurface oxygen is produced at 470 K [31]. For stepped Rh surfaces, the activation energy of subsurface oxygen formation is lower than for close-packed planes such as Rh(111) [32, 33], thus subsurface oxygen formation with an observable rate may occur at lower temperature.

In order to verify the proposed model and, in particular, the feedback mechanism, the observed oscillations were analyzed by a microkinetic model [30]. The model predicts a critical dependence of the oscillation frequency on the activation energy of subsurface oxygen formation, suggesting a feedback loop between the rate of oxygen adsorption and the concentration of subsurface oxygen. The frequency of oscillations sensitively depends on the rate of subsurface oxygen formation and its depletion, both governed by the activation energy of subsurface oxygen formation. The more easy the subsurface oxygen is formed, the higher the predicted oscillation frequency is, which is indeed observed in the experiments: the more “stepped” and “kinked” the surfaces are (Fig. 5g–j), the higher the oscillation frequency is. This model also explains the observations of field-induced oscillations on Rh nanotips [34, 35]. As shown earlier [36], a high electric field lowers the activation energy for surface oxidation of Rh, enabling oscillations under HV conditions. Again, the surface structure library in the form of a polycrystalline Rh foil with highly-indexed stepped surfaces enabled to discover a new phenomenon.

## Particle Size Libraries

Applied catalysis frequently utilizes oxide supported nm-sized metal particles, with marked effects of particle size on atomic and electronic structure, abundance of specific surface facets, defects, etc. In such reaction systems of limited dimensions, differently oriented nanofacets and communication effects (arising from the coupling of the kinetics of different regions) often play a critical role [37, 38]. Another more general (but often ignored) size effect arising from nm-dimensions, is the influence of reaction-induced fluctuations [39, 40]. For example, for SiO_2_ supported Pd nanoparticles, fluctuations were made responsible for the vanishing bistability in CO oxidation, upon decreasing particle size. This effect, theoretically predicted by Fichthorn et al. [41] and first experimentally observed by Suchorski et al. [39], may strongly affect the parameter range of catalyst activity [42]. This once more stresses the importance of studies of particle size effects in catalysis.

**Fig. 6:**
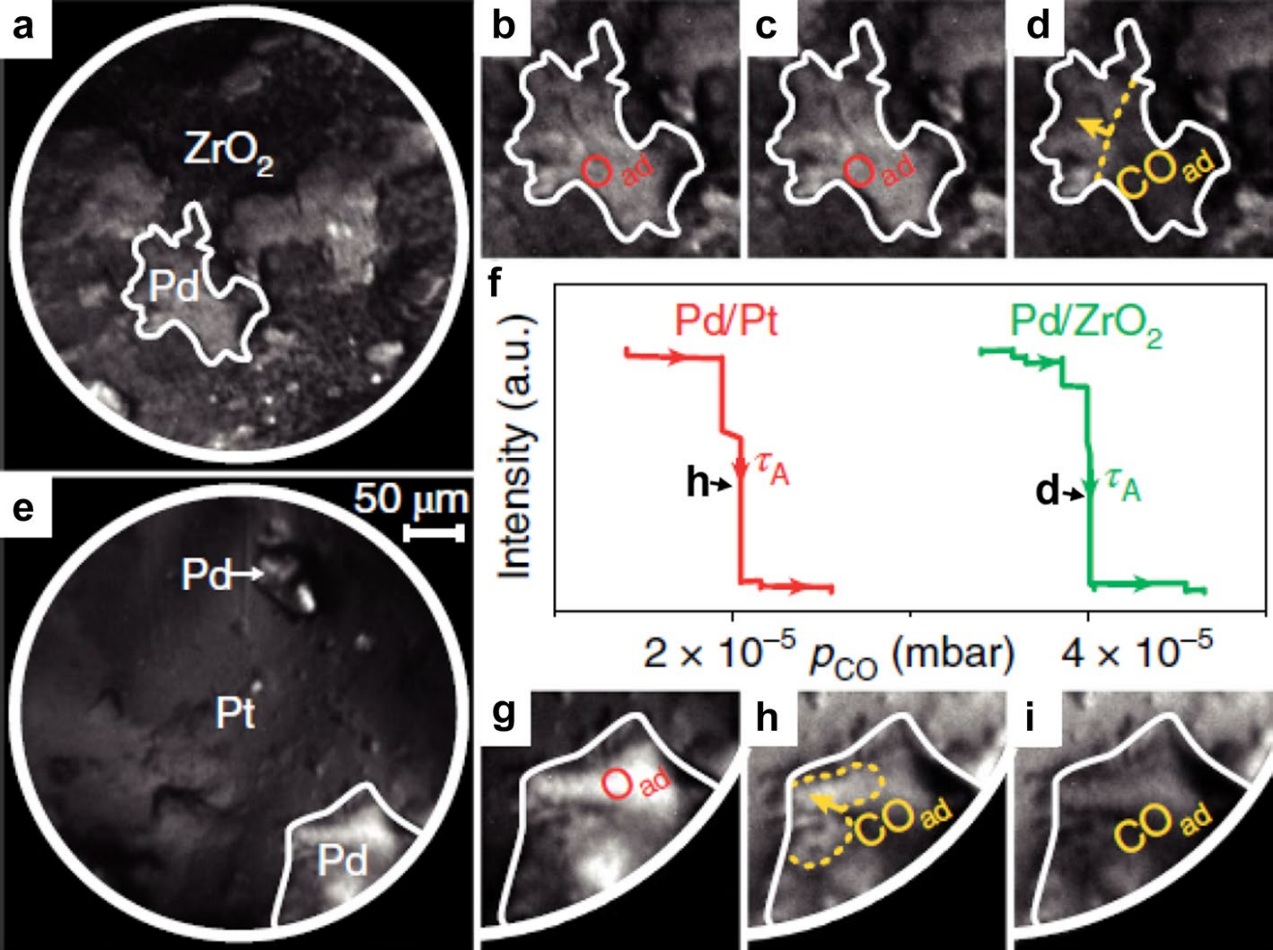
Long-range effect of the metal/oxide interface on CO oxidation on Pd: **a** PEEM field of view showing Pd agglomerates supported on ZrO_2_; **b, c** Pd agglomerate marked in (**a**) in the active steady state (oxygen covered surface) during increasing the CO pressure up to 4 × 10^−5^ mbar at constant T = 473 K and p_O2_ = 1.3 × 10^−5^ mbar; **d** isothermal kinetic transition (propagation of the CO front) to the inactive steady state at p_CO_ = 4 × 10^−5^ mbar; **e** Pd agglomerates of similar size as in (**a**), but supported by Pt, **f** local PEEM intensity of the oxide supported Pd agglomerate marked in (**a**) (green curve) and of the Pt supported Pd marked in (**e**) (red curve); **g** Pd agglomerate marked in (**e**) being in the active state at the same T and p_O2_ as in (**b**–**d**) and at p_CO_ < 2 × 10^−5^ mbar. **h** Kinetic transition to the inactive state (CO front propagates) at p_CO_=2 × 10^−5^ mbar; **i** Pd agglomerate in the inactive state (CO covered) at p_CO_ > 2 × 10^−5^ mbar. Under these conditions, the oxide supported Pd agglomerate (**c**) still remains active (oxygen covered). Reproduced from [38] with permission from Springer Nature

Thus, a spatially-resolving device such as PEEM may be very valuable for studies of size- and communication-effects in catalysis, if a collection of particles of different size and shape can be simultaneously imaged within the field of view. In analogy to polycrystalline foils representing many differently oriented domains (structure libraries), collections of various particle sizes (size libraries) can help to replace averaging studies of many samples with identical or at least similar particle size (note that the preparation of truly monodisperse samples is right difficult). Using a size library, many particles of different size can be simultaneously monitored during the reaction and size effects can be directly discerned (see the idea of the experiment in Fig. 3b). To prove the feasibility of such an approach, we fabricated a size library by pressing metal powder (“Pd black” containing µm-sized Pd particles) onto thin oxide films (see SI in Ref. [38]). Already the first application of such a library allowed to detect a long-range communication effect for Pd particles supported by catalytically rather inert oxides (ZrO_2_ or Al_2_O_3_) (Fig. 6 [38]).

**Fig. 7:**
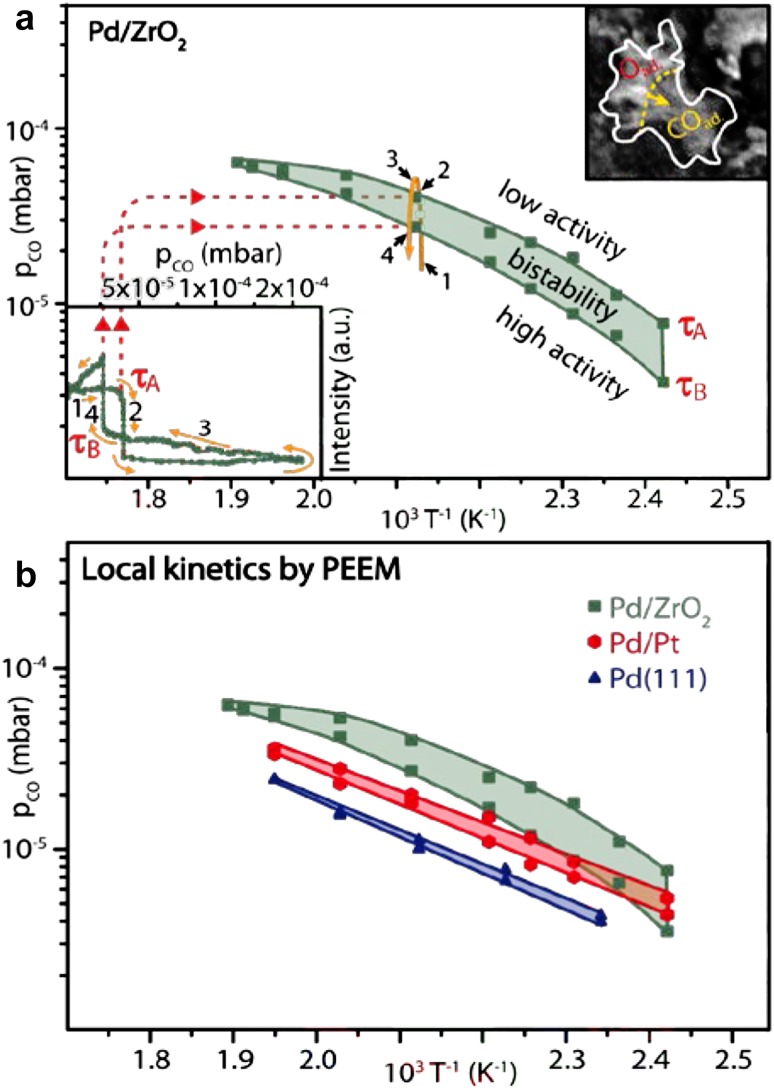
Local kinetic data for CO oxidation on Pd. **a** Kinetic phase diagram for CO oxidation on the individual Pd agglomerate supported by ZrO_2_ marked in Fig. 6a, measured by PEEM at p_O2_ = 1.3 × 10^−5^ mbar. The left-hand inset displays the PEEM intensity hysteresis during a cycle-wise variation of the CO pressure at 473 K. The right hand inset shows the chosen Pd agglomerate during the kinetic transition τ_B_ from the inactive to the active steady state; **b** catalytic behaviour of Pd supported on ZrO_2_, Pd supported on Pt and of a Pd(111) domain of a polycrystalline Pd foil. Reproduced from [38] with permission from Springer Nature

Kinetic transitions, initiating along the nm-narrow metal/oxide interface, influenced the kinetics of an entire µm-sized Pd particle (i.e. of sites thousands of nm away from the interface). This led to a better CO tolerance of a whole cm-sized sample with hundreds of Pd particles, as confirmed by (macroscopic) averaging mass spectroscopy. It is also evident from the kinetic phase diagrams for supported and unsupported Pd (Fig. 7): the diagrams for the oxide-supported Pd are shifted to higher CO pressures in comparison to Pt-supported Pd or the Pd(111) surface.

**Fig. 8:**
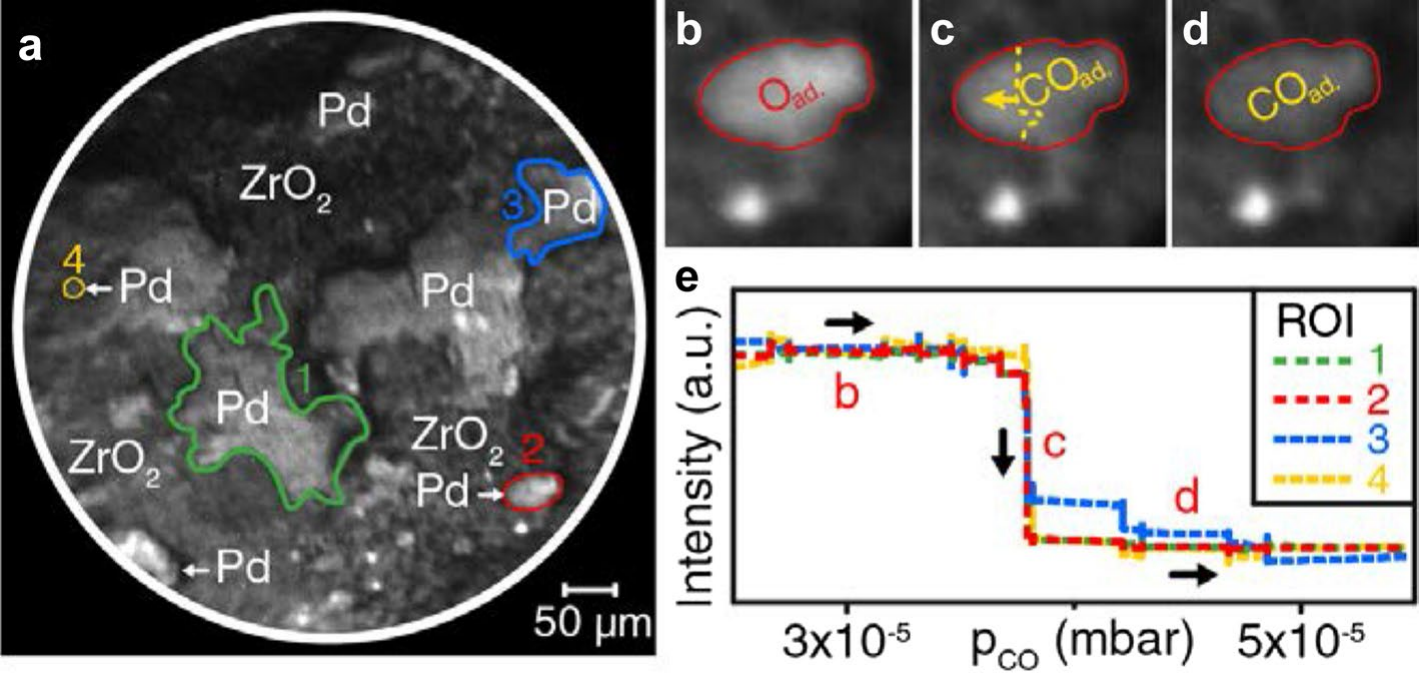
CO oxidation on ZrO_2_ supported Pd aggregates: **a** PEEM field of view with four Pd aggregates of significantly differing size marked by green, red, blue and yellow colour lines; ** b**–**d** CO front propagation on the Pd agglomerate no. 2, **e** direct comparison of the normalized PEEM intensity reflecting the kinetic transition for the four Pd aggregates marked in (**a**). Reproduced from SI of Ref. [38] with permission from Springer Nature

It is apparently important to examine whether this remarkable effect depends on the size and shape of the Pd particles, a task which can be performed by in situ PEEM. Figure 8 shows how four differently sized Pd particles undergo a kinetic transition from the active to the inactive state. Evaluation of the local PEEM intensity of the four different particles (Fig. 8e) [38] demonstrates that the transitions occur simultaneously, i.e. in the size range of 1–200 µm there was no particle size effect. Of course, in case of µm-sized particles, a size effect is not expected, but modern PEEM devices with nm-resolution can study size effects, applying the described approach to nm-sized particles, similar to those used in commercial catalysts. Again, these findings were only made possible using a *size and shape library* of catalytically active Pd particles, combined with spatially-resolved kinetic techniques, enabling the evaluation of local kinetics for each individual Pd particle in the field of view.

## “Curved” Crystals

As mentioned, apart from polycrystalline materials, a surface structure library can also be created by “curved” surfaces, e.g. cylindrical crystals. Already in 1992, a cylindrical Pt crystal exhibiting all orientations of the [001] zone was utilized to examine the structure dependence of CO oxidation [43]. However, thermal inertia of bulky crystals and difficulties in the preparation of atomically clean surfaces in UHV are factors limiting the application of “fully cylindrical” samples. As a compromise, a cylinder section has often been used, but with corresponding reduction in the number of available structures [44].

One can also vary the crystal curvature in two orthogonal directions, forming “dome-shaped” surfaces, which provide a wider selection of different crystallographic surfaces and directions. However, such samples almost always have a small opening angle (< 10°–12°), which limits the number of accessible crystallographical directions [45]. Accordingly, it would be favorable to use a curved crystal with a large (> 45°) opening angle which provides a wide range of crystallographic orientations. Such tip-shaped curved crystals, with a nearly hemispherical apex, but of nm-dimensions, are used as probing tips in scanning probe microscopies (scanning tunneling and atomic force). In field emission based microscopies [field electron microscopy (FEM) and field ion microscopy (FIM)] such tips have been used as samples themselves, also serving as models of individual catalytic nanoparticles [46]. However, the apexes of such nanotips exhibit individual facets of only a few nanometers in size, which makes surface analysis very difficult [47].

**Fig. 9:**
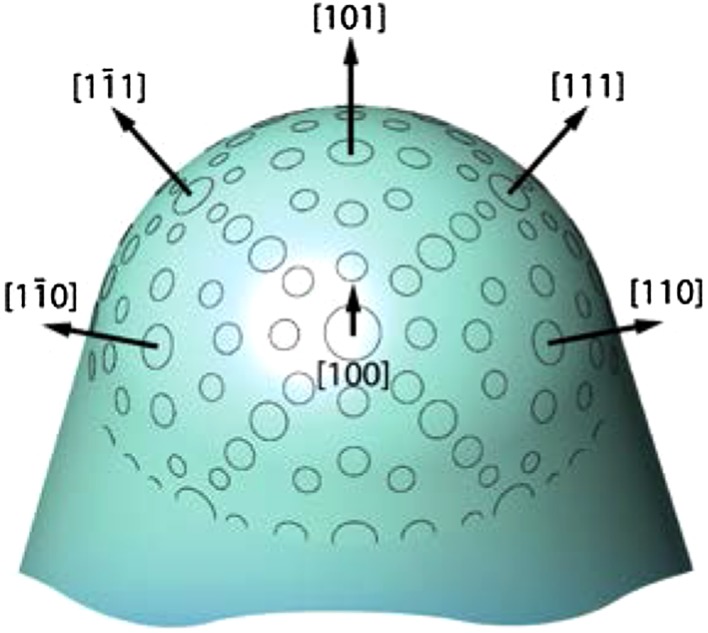
A schematics of a µm-sized curved crystal with large opining angle of a precious metal (e.g. Pt, Pd, Rh) exposing a wide range of differently oriented facets on its apex surface

A similar, but more “blunt”, metal tip with a curvature in the µm-range rather than in the nm-range, in fact represents a curved crystal with a large opening angle (Fig. 9). The geometry of the surface of the blunt tip can be visualized with a scanning electron microscope, and its crystallography can be determined by means of stereographic projection. Such a well-defined precious metal tip can then serve as another type of surface structure library for model catalysis. FIM (with atomic resolution) cannot be applied to such crystals, due to the large radius of tip curvature [46, 47], but FEM (spatial resolution ~ 2 nm) is well-suited as spatially-resolved examination method. Since FEM, like PEEM, is a work function based microscopy (with a certain influence of the local field distribution), the concept of kinetics by imaging can be applied and first results for H_2_ oxidation on Rh were already obtained [48].

### Résumé and Outlook

The difficulty of conventional comparative catalytic studies of surface structure and particle size effects, á la “one (uniform) sample after the other”, can be favourably overcome by using “surface structure and size libraries” in combination with spatially-resolved kinetic methods. In this approach, different crystallographic orientations or different particle sizes are simultaneously exposed to identical reaction conditions. Polycrystalline precious metal foils (e.g. Pt, Pd, Rh), consisting of many µm-sized domains of different structures, have enabled new interesting findings in the field of catalytic ignition, reaction front propagation and oscillating surface reactions. Similarly, spatially-resolved studies of oxide supported µm-scaled Pd particles of various sizes, distributed over the surface of *one* sample (size library) have revealed novel long-ranging metal/oxide interface effects. To complete the set, µm-sized “curved” crystals represent another type of “surface structure library”, promising further fascinating insights into structure dependency on the route towards better understanding catalytic heterogeneity.

In the present contribution, we mainly focused on PEEM studies of CO or H_2_ oxidation on individual domains of polycrystalline Pt, Pd and Rh foils and on the catalytic behaviour of supported and unsupported metal particles. However, the above approach is not limited to these two surface reactions and to PEEM imaging. Once a surface process can be visualized in situ by an imaging technique (e.g. by low-energy electron microscopy (LEEM [49, 50]), ellipso-microscopy for surface imaging [51], reflection anisotropy microscopy [51], infrared and Raman imaging [52], metastable impact electron emission microscopy ([53]) etc.), the concept of kinetics by imaging can be applied, provided a relation between the local image intensity and kinetic parameters can be identified. Particularly LEEM, with its possibilities to combine real time imaging and gaining local structure information via micro-diffraction [54, 55], seems promising in this aspect. Catalytically relevant surface processes, such as the growth of two-dimensional layers/islands of graphene [56] or cerium oxide [57], or even catalytic reactions in the nanospace confined between substrate and overlayer [58, 59], have been examined by this technique.

Spatially-resolved methods, despite not working in parallel mode, are also powerful, e.g. photoemission [60], tip-enhanced Raman [61], fluorescence [62, 63] and X-ray absorption/emission [64] microspectroscopies. Using them and the kinetics by imaging approach, fascinating studies of surface structure and particle size libraries are to be expected.
